# Gold nanoparticles induce cytotoxicity in the alveolar type-II cell lines A549 and NCIH441

**DOI:** 10.1186/1743-8977-6-18

**Published:** 2009-06-22

**Authors:** Chiara Uboldi, Daniele Bonacchi, Giada Lorenzi, M Iris Hermanns, Christine Pohl, Giovanni Baldi, Ronald E Unger, C James Kirkpatrick

**Affiliations:** 1 Institute of Pathology – REPAIR Lab, University Medical Center of the Johannes Gutenberg University Mainz, Langenbeckstrasse 1, 55101 Mainz, Germany; 2 CERICOL – Centro di Ricerche Colorobbia, Via Pietramarina 123, 50059 Firenze, Italy

## Abstract

**Background:**

During the last years engineered nanoparticles (NPs) have been extensively used in different technologies and consequently many questions have arisen about the risk and the impact on human health following exposure to nanoparticles. Nevertheless, at present knowledge about the cytotoxicity induced by NPs is still largely incomplete. In this context, we have investigated the cytotoxicity induced by gold nanoparticles (AuNPs), which differed in size and purification grade (presence or absence of sodium citrate residues on the particle surface) *in vitro*, in the human alveolar type-II (ATII)-like cell lines A549 and NCIH441.

**Results:**

We found that the presence of sodium citrate residues on AuNPs impaired the viability of the ATII-like cell lines A549 and NCIH441. Interestingly, the presence of an excess of sodium citrate on the surface of NPs not only reduced the *in vitro *viability of the cell lines A549 and NCIH441, as shown by MTT assay, but also affected cellular proliferation and increased the release of lactate dehydrogenase (LDH), as demonstrated by Ki-67 and LDH-release assays respectively. Furthermore, we investigated the internalization of AuNPs by transmission electron microscopy (TEM) and we observed that particles were internalized by active endocytosis in the cell lines A549 and NCIH441 within 3 hr. In addition, gold particles accumulated in membrane-bound vesicles and were not found freely dispersed in the cytoplasm.

**Conclusion:**

Our data suggest that the presence of contaminants, such as sodium citrate, on the surface of gold nanoparticles might play a pivotal role in inducing cytotoxicity *in vitro*, but does not influence the uptake of the particles in human ATII-like cell lines.

## Background

Nanoparticles (NPs) are generally defined as having a diameter below 100 nm and they are believed to display different properties compared to their bulk material [1-4]. NPs are currently being used extensively in different industrial technologies, in biomedical applications [5-7] and in cosmetics [8]. The main route of exposure to NPs is the respiratory tract, but the human organism can come into contact with particles via ingestion, deposition and injection. The epithelial surface area of a human lung, which is approximately 140 m^2 ^[9], interacts with an enormous number of inhaled nanosized materials with each breath. Inhaled nanoparticles are then deposited along the respiratory tract depending on their size: discrete inhaled nanoparticles preferentially accumulate in the nasal region, while smaller NPs are able to reach the deep lung, inducing inflammation and the formation of reactive oxidative species in the alveolar region [10-12]. In addition, the alveolar region, which is composed of many different cell types in close association to the endothelium and therefore connected to the blood circulation, is considered to be a privileged site for NPs deposition and translocation. It has been shown that after being internalized NPs are able to escape their site of deposition, entering the blood or the lymphatic circulation and redistributing to other organs, such as the liver and the central nervous system [13-16]. Thus, for example, in rats inhaled ultrafine titanium dioxide NPs have been shown to be taken up and rapidly redistributed from the lung compartment to the circulation [17-19]. In humans, inhaled carbon nanoparticles were reported to translocate from the lung tissue into the blood stream and become distributed in extrapulmonary sites [20]. However, due to the complexity of the pulmonary system, as well as the increasing number of NPs released in the environment, a systematic evaluation of nanoparticles-mediated cytotoxicity has not as yet been possible.

Currently gold nanoparticles (AuNPs) are used in different biomedical applications, such as intracellular gene regulation [21], chemotherapy [22] and drug delivery [5,6], as well as in optical and electronic applications [23]. Investigations on the *in vitro *cytotoxicity of gold nanoparticles have already been performed, but so far results were inconclusive. AuNPs were shown to induce cytotoxicity in Cos-1 cells [24] and in human dermal fibroblasts [25], but not in human leukemic cells [26] or murine macrophages [27], where particles were taken up and stored in intracellular perinuclear vesicles. In addition, it is still unknown whether the size or the surface coating of the gold nanosized particles induces cellular damage and cytotoxicity *in vitro*. In this context, Pan et al. [28] have shown that the size of the particles plays a pivotal role in inducing cytotoxicity in HeLa cells. They have demonstrated that small AuNPs (1–2 nm size), but not the larger (15 nm size), could induce toxicity in HeLa cells. On the other hand, Niidome et al. [29] have shown that the toxic potential is triggered by the surface modification of the gold nanoparticles. In fact, bromide-stabilized gold nanorods induced severe cytotoxicity in HeLa cells, whereas PEG-modified gold particles, which displayed a neutral surface, could only induce moderate toxicity. Nevertheless, Connor et al. [26] demonstrated that neither the surface characteristics nor the size of gold nanoparticles seemed to play a role in inducing cytotoxicity in the human leukemic cell line K562. In fact, they indicated that gold nanoparticles with different size and surface modifications could be internalized by the cells but did not exert cytotoxicity. On the basis of this the authors proposed that the shape of the NPs, and eventually intracellular modifications of the nanoparticles determined by the cellular environment, could be responsible for causing toxicity.

Due to the contradictory results of the previous investigations, the present study was aimed at examining the effect of gold nanoparticles in the deep respiratory tract. To this end, the human alveolar type-II (ATII)-like cell lines A549 and NCIH441 were exposed to AuNPs with different diameter (9.5 – 25 nm) and purification grade (presence or absence of an excess of sodium citrate), and the toxicity induced was evaluated by classical cytotoxicity assays. Briefly, we demonstrated that gold nanoparticles with an excess of sodium citrate impaired the viability, the membrane integrity and the proliferation of the human ATII-like cell lines A549 and NCIH441 *in vitro*. As a result, we then examined whether the non-toxic potential of the gold nanoparticles without sodium citrate was due to an impaired uptake of the particles. By transmission electron microscopy (TEM) we observed that AuNPs were indeed internalized by the ATII-like cell lines A549 and NCIH441, independent of the presence or absence of contaminants on the particle surface, and independent of the size and the shape of the particles.

## Results and Discussion

### Synthesis and characterization of gold nanoparticles

**Figure 1:**
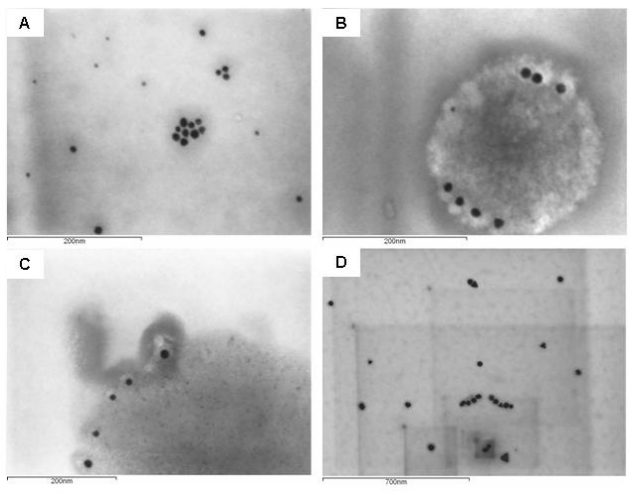
**Characterization of the gold nanoparticles using scanning transmission electron microscopy**. Water dispersed gold nanoparticles were analyzed by scanning transmission electron microscopy (STEM) to determine their size, shape and distribution. AuS0302 (A), AuS0302-RIT (B), AuS0302-RIS02 (C) and AuS0302-RIS04 (D) appeared monodispersed in solution or formed small aggregates, and their diameter ranged between 5 nm (A) and 25 nm (D). With the exception of the sample AuS0302-RIS04, all particles synthesized displayed a spherical shape.

Water-dispersed gold nanoparticles (AuNPs) were synthesized by wet chemical synthesis based on the reduction of tetrachloroauric acid. Three AuNPs batches were produced (AuS0302; AuS0302-RIS02; AuS0302-RIS04) and consequently analyzed to identify their physicochemical properties. Dynamic light scattering (DLS) indicated that the batch AuS0302 had a large dispersion of the particles (polydispersity index PDI ≥ 0.3), with an average volume-weighted diameter of 21.42 nm (Table 1). The hydrodynamic diameter was not modified, although AuNPs underwent purification and dialysis to reduce the excess of sodium citrate retained during the synthesis, and indeed the hydrodynamic diameter increased for the samples AuS0302-RIS02 and AuS0302-RIS04, suggesting that the experimental procedure adopted to produce particles with increasing diameter was successful (Table 1). Particles were further analyzed by scanning transmission electron micrographs (STEM). As shown in Figure 1, particles did not form large aggregates and they were quite uniformly dispersed. Interestingly, STEM revealed that the gold nanoparticles had a smaller diameter and narrower size distribution compared to the results obtained by DLS (Table 1). In fact, over 200 AuNPs were analyzed, and the statistical distribution revealed a mean diameter of 9.5 nm, 11.19 nm and 25.0 nm for AuS0302, AuS0302-RIS02 and AuS0302-RIS04 respectively. As large PDI were observed, it was not possible to convert the volume-weighted diameter from dynamic light scattering measurement to the number-weighted diameter calculated from electron microscopy images. In this case electron microscopy gives more reliable results, as it is a direct measurement of the diameter and confirms that the proposed synthetic method is effective in producing nanoparticles of increasing size.

**Table 1:** Physicochemical characterization of the gold nanoparticles.

**Samples**	**Solvent**	**%Au w/w**	**PDI**	**dV mean (nm)**	**D STEM (nm)**
**AuS0302**	H_2_O	0.259	0.362	21.42	9.44
**AuS0302-RIT**	H_2_O	0.256	0.330	21.20	9.79
**AuS0302-RIS02**	H_2_O	0.282	0.331	27.17	11.19
**AuS0302-RIS04**	H_2_O	0.288	0.362	28.18	25.0

Water-dispersed gold nanoparticles were produced by wet chemical reaction based on the reduction of tetrachloroauric acid. This technique allowed the synthesis of nanoparticles with increasing diameter, as shown by DLS and STEM.

PDI: polydispersity index

dV: mean diameter calculated by dynamic light scattering (DLS)

D STEM: scanning transmission electron micrographs

To remove the excess of sodium citrate retained during the synthesis, the sample AuS0302 underwent filtration and dialysis, and the purified product was identified as AuS0302-RIT. The physicochemical properties of the particles were preserved during tangential flow filtration (Table 1), and this procedure did not induce nanoparticle agglomeration, as was shown by STEM (Figure 1). Analysis of the dried permeate by differential scanning calorimetry – thermogravimetry (DSC-TG) revealed that by filtration nearly 92% of sodium citrate was removed from the final filtrate solution AuS0302-RIT (data not shown). Nevertheless, the presence of PVP residues on the surface of the gold nanoparticles cannot be excluded. In addition, possible effects on the particles surface following dialysis were investigated by measuring the Zeta potential of the particles, revealing that the Zeta potential before (-13 ± 2 mV) and after (-12 ± 2 mV) dialysis did not change.

### Cytotoxicity of gold nanoparticles

#### MTT assay

**Figure 2:**
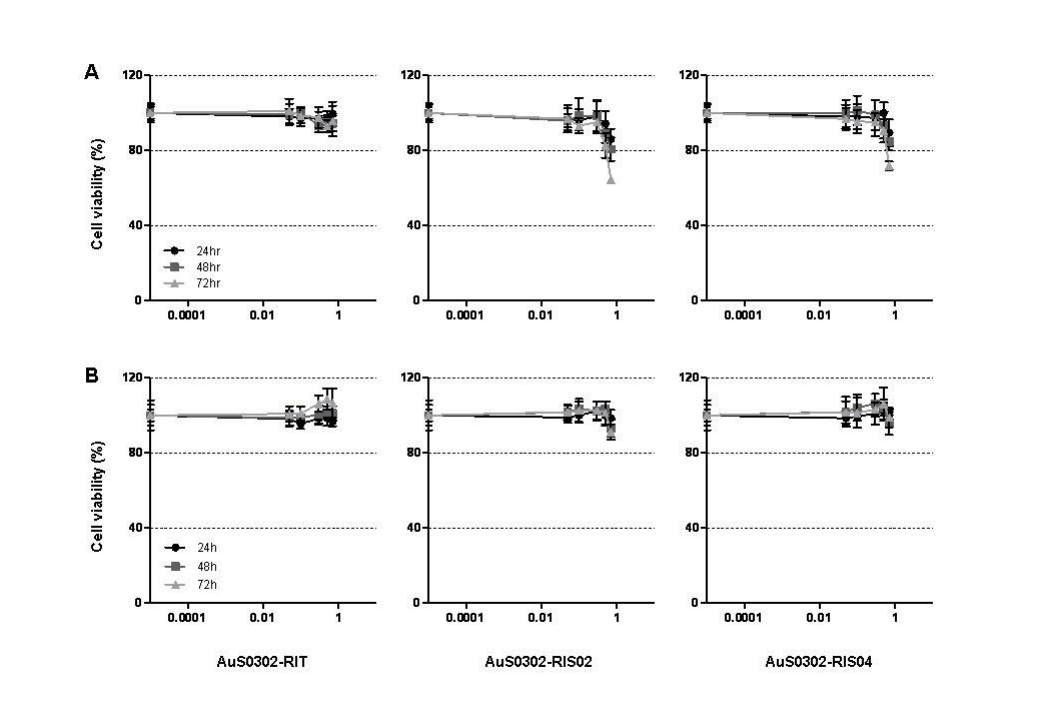
**Comparison of the viability of the cell lines A549 and NCIH441 exposed to gold nanoparticles**. The cell lines A549 (A) and NCIH441 (B) in the logarithmic growth phase were exposed to AuNPs for 24, 48 and 72 hr. The cell viability was measured by the MTT assay as described in the Experimental Section. Each result represents the mean viability ± standard deviation (SD) of three independent experiments and each of these was performed in triplicate. Cell viability was calculated as the percentage of the viable cells compared to the untreated controls.

To examine the cytotoxicity of gold nanoparticles (AuNPs), monocultures of the human alveolar type-II-like cell lines A549 and NCIH441 were incubated with increasing amounts of AuNPs for 24–72 hr, and the cell viability, expressed as percentage of the untreated control (100% cell viability), was investigated by 3-(4,5-dimethylthiazol-2-yl)-2,5-diphenyltetrazolium bromide (MTT) assay. Figure 2A shows that AuS0302-RIT nanoparticles did not have any effect on the viability of the human ATII-like cell line A549, while AuS0302-RIS02 and AuS0302-RIS04, which present an excess of sodium citrate on their surface, only exerted a mild toxicity at high concentrations (0.7 mM). Even after 72 hr continuous exposure to Au/sodium citrate nanoparticles, 60–70% cell viability could be detected. Interestingly, as shown in Figure 2B, by the MTT assay gold-induced cytotoxicity in monoculture of the human ATII-like cell line NCIH441 could not be detected. Our data are consistent with previous investigations in which citrated and biotinylated gold nanoparticles were shown to be non-toxic in the human leukemia cell line K562 [26] and colloidal and lysine-capped AuNPs did not exert any effect in the mouse macrophage cell line RAW264.7 [27]. Connor [26] and Shukla [27] could not detect any decrease in the cell viability after exposing cells to nanoparticles presenting citrate, biotin and borohydride as surface modification, and, as shown by MTT assay, after 72 hr exposure the human leukemia cell line K562 and the mouse macrophage cell line RAW264.7 were still viable.

#### Ki-67 assay

**Figure 3:**
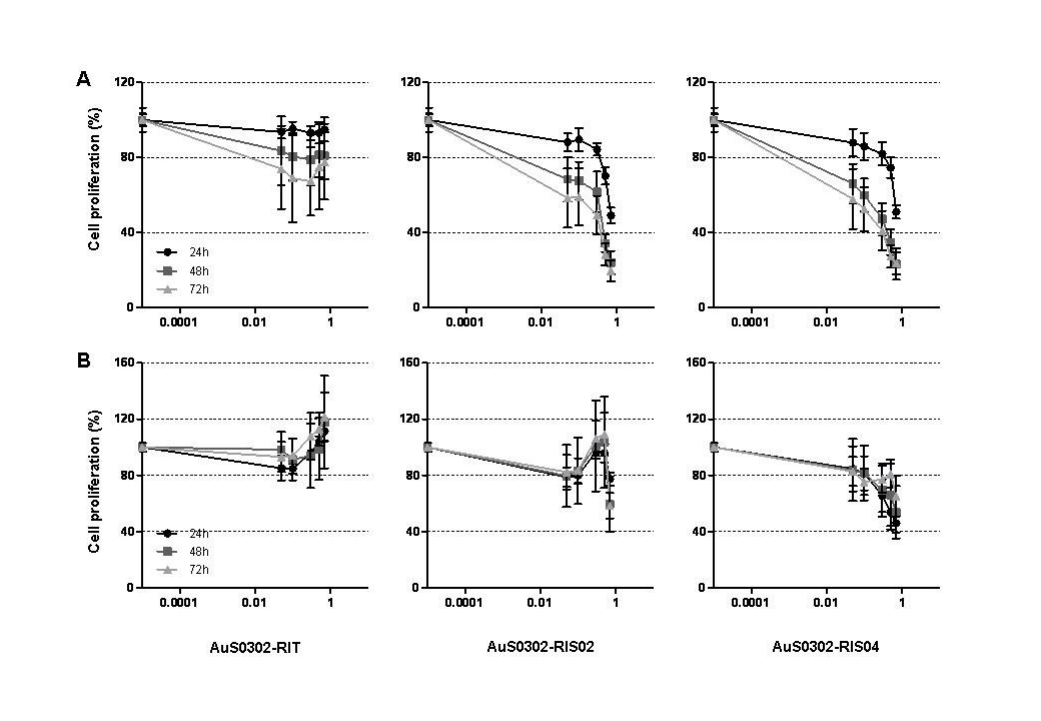
**Comparison of the proliferation of the cell lines A549 and NCIH441 exposed to gold nanoparticles**. The cell lines A549 (A) and NCIH441 (B) in the logarithmic growth phase were exposed to AuNPs for 24, 48 and 72 hr. Each result represents the mean proliferation ± standard deviation (SD) of three independent experiments and each of these was performed in triplicate. Cell proliferation was calculated as the percentage of the viable cells compared to the untreated controls.

The gold-mediated cytotoxicity on the human ATII-like cell lines A549 and NCIH441 was further investigated by Ki-67 assay for cell proliferation. As for the MTT assay, cells were exposed to AuNPs for 24–72 hr. As shown in Figure 3A, when exposing the ATII-like cell line A549 to citrated nanoparticles AuS0302-RIS02 and AuS0302-RIS04 a dose- and time-dependent impairment of proliferation was observed. Compared to the untreated control, after 24 hr the proliferation of the cell line A549 was reduced by about 50%, and following longer incubation periods (48–72 hr) only 20–30% proliferation could be measured in the presence of gold nanoparticles. AuS0302-RIS02 and AuS0302-RIS04 exerted a milder effect on the proliferation of the human ATII-like cell line NCIH441 (Figure 3B), similar to that observed in the MTT assay. After 24, 48 or 72 hr exposure to gold nanoparticles with an excess of sodium citrate, 40–60% proliferating NCIH441 cells could be detected, and the same pattern could be observed upon comparing the different exposure periods. The results obtained from exposing the human ATII-like cell lines A549 and NCIH441 to the gold nanoparticles AuS0302-RIT were of special interest. AuS0302-RIT, which contained a reduced amount of sodium citrate as surface contaminant, slightly impaired the proliferation of the ATII-like cell line A549: only a 20–30% reduction in the proliferation of the cell line A549 was measured at 48–72 hr incubation with AuS0302-RIT (Figure 3A). Similarly, the cell line NCIH441 did not show any reduced proliferation after 24–72 hr incubation with AuS0302-RIT nanoparticles (Figure 3B). Taken together, the proliferation of the cell line NCIH441 was less affected by the presence of gold nanoparticles compared to the ATII-like cell line A549, and in general the presence of sodium citrate appeared to lead to a reduction in the proliferation of the ATII-like cell lines A549 and NCIH441 *in vitro*.

#### Lactate dehydrogenase (LDH) release assay

**Figure 4:**
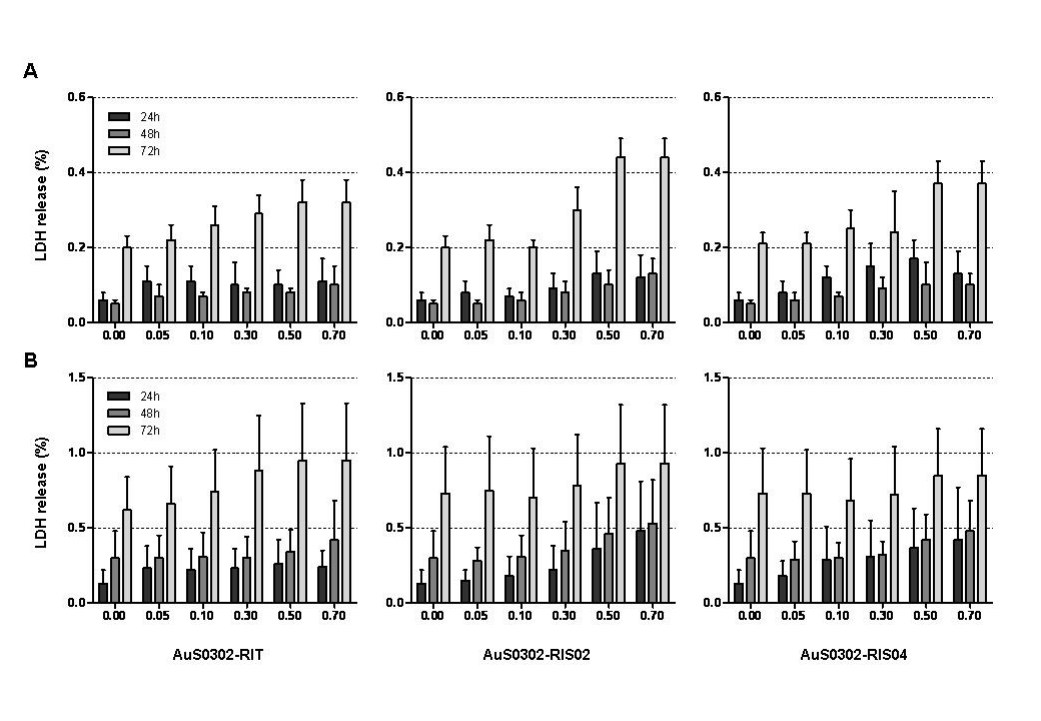
**Comparison of the cytotoxicity induced by gold nanoparticles in the cell lines A549 and NCIH441: lactate dehydrogenase release**. The cell lines A549 (A) and NCIH441 (B) in the logarithmic growth phase were exposed to AuNPs for 24, 48 and 72 hr. The gold-induced cytotoxicity was measured by LDH release assay. Each result represents the mean LDH release ± standard deviation (SD) of three independent experiments and each of these was performed in triplicate.

To further investigate the possible cytotoxicity induced by gold nanoparticles on human type-II-like pneumocytes *in vitro*, the cell lines A549 and NCIH441 were exposed to gold nanoparticles and the amount of LDH released, which is a sensitive and accurate marker for cellular toxicity, was evaluated. Figure 4A clearly shows that after 24–48 hr exposure gold nanoparticles induced a mild LDH release in the human ATII-like cell line A549, independent of the presence or absence of surface contaminants. In addition, after 72 hr exposure to AuNPs a dose-dependent release of lactate dehydrogenase in the supernatant was observed and the amount of LDH released was significantly higher compared to shorter exposure times (24–48 hr) (Figure 4A). Interestingly, we observed a comparable LDH release after 24–48 hr treatment to 0.7 mM AuNPs, and the lactate dehydrogenase release increased significantly after 72 hr continuous exposure. The same results were obtained when the cell line NCIH441 was exposed to gold nanoparticles. As shown in Figure 4B, after 24–48 hr exposure, the amount of LDH was significantly lower compared to the 72 hr time point and, as already observed in the cell line A549, the release of LDH after 72 hr exposure was dose-dependent. Interestingly, in the presence of gold nanoparticles the cell line NCIH441 released a higher amount of LDH compared to the cell line A549. As for the cell line A549, the presence of an excess of sodium citrate on the particle surface increased the secretion of LDH in the supernatants of the cell line NCIH441 after 72 hr exposure.

To summarize, the presence of sodium citrate on the surface of gold nanoparticles reduced the viability (as shown by MTT assay) and impaired the proliferation (as demonstrated by Ki-67 assay) in both the human ATII-like cell lines A549 and NCIH441. In addition, the results obtained by MTT and Ki-67 assay suggest that the cell line NCIH441 is slightly more resistant to the AuNPs-induced cytotoxicity compared to the cell line A549. However, our data indicate that it is the presence of contaminants, such as sodium citrate, and not the size of the particles that impairs the viability and the proliferation of the cell lines A549 and NCIH441. These results are consistent with previous investigations performed with dermal fibroblasts [25] and leukemic cells [26]. Pernodet et al. [25] demonstrated that gold/citrate nanoparticles impaired the proliferation of dermal fibroblasts and induced an abnormal formation of actin filaments, causing therefore a reduced cellular motility and influencing the cell morphology. On the contrary, Connor et al. [26] reported that citrated and biotinylated 18 nm AuNPs did not induce toxicity in leukemic cells (cell line K562), whereas smaller particles were much more toxic. In addition, gold nanoparticles did not exert any toxic effect, as shown by the MTT assay, in the human gastrointestinal cancer cells Panc-1 and HepB3 [30], nor in HeLa cells [27,31]. Hauck et al. [31] have demonstrated that the use of different surface coatings does not influence the toxicity induced by gold nanoparticles in HeLa cells, but it was of extreme importance to tune the uptake of AuNPs. In addition, Hauck et al. [31] suggest that the lack of toxicity was due to the fact that AuNPs were stored intracellularly in membrane-bound vesicles and therefore particles could not directly interfere with the nuclei or with other cytoplasmic organelles and induce toxic events. Nevertheless, there is evidence that the size of the gold nanoparticles induces *in vitro *cytotoxicity in HeLa cells. In fact, Pan et al. [28] have shown that the size, and not the particle chemistry, is responsible for determining the toxicity of the gold particles. After exposing for 48 hr the cells to increasing concentrations of nanosized gold particles, by MTT assay Pan et al. observed that Au clusters of different sizes had a different toxic potential. In addition, they demonstrated that on using different particles stabilizers, the toxicity observed was almost indistinguishable, leading the authors to conclude that it is the size of the particles to play a pivotal role in inducing *in vitro *cytotoxicity.

Citrate is commonly used as reducing agent and its presence on the surface of gold nanoparticles might interfere with the MTT assay, thus leading to an overestimation of the viability data and to the conclusion that AuNPs exert only a mild toxicity or, in some cases, no toxicity at all in the cell lines A549 and NCIH441. Consequently, the cytotoxicity induced by AuNPs was further investigated by Ki-67 and LDH release assay. By Ki-67 assay it has been shown that the sodium citrate-nanoparticles AuS0302-RIS02 and AuS0302-RIS04 impaired (up to 50–70% reduction) the proliferation of the cell lines A549 and NCIH441. A slightly reduced proliferation was also measured following exposure to the sample AuS0302-RIT, which presents a significantly reduced amount of sodium citrate as surface contaminant. Investigating the amount of lactate dehydrogenase (LDH) released upon exposure to AuNPs we have shown that the major effects are detectable after 72 hr treatment. In addition, in the cell lines A549 and NCIH441 a dose-dependent LDH release was observed. Summarizing, we demonstrated the cytotoxic potential of gold nanoparticles in alveolar type-II-like cell lines using different assays (MTT, Ki-67 and LDH release) and different end-points (cell viability, cell proliferation and membrane integrity).

### Internalization of gold nanoparticles

**Figure 5:**
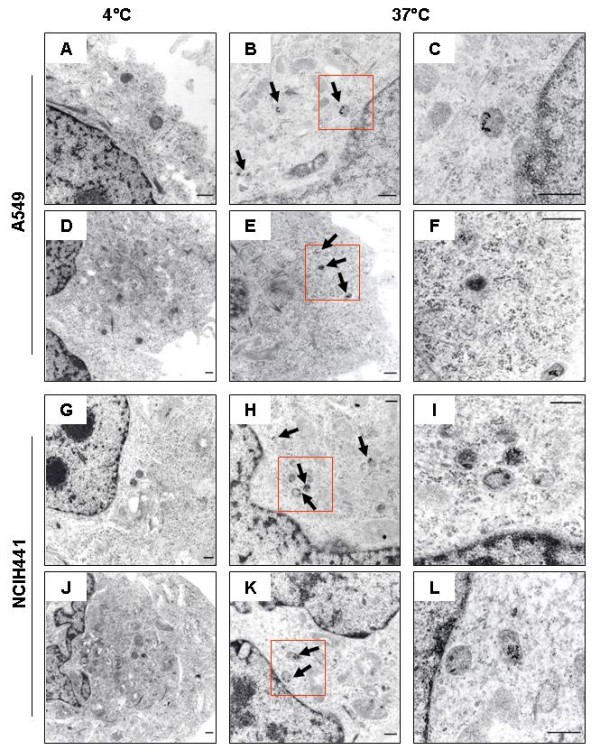
**Internalization of gold nanoparticles in the cell lines A549 and NCIH441**. The human ATII-like cell lines A549 and NCIH441 were incubated in the presence of AuS0302-RIT and AuS0302-RIS02 nanoparticles. After 3 hr incubation, cells were fixed and analyzed by transmission electron microscopy (TEM). At 37°C, 0.3 mM AuS0302-RIT (E-F and I-J) and AuS0302-RIS02 (G-H and K-L) were taken up by the cell lines A549 and NCIH441. Gold nanoparticles were observed localized in intracellular membrane-bound vesicles and not freely dispersed in the cytoplasm. In parallel, cells were treated with ice-cold nanoparticle solutions and incubated at 4°C for 3 hr. None of the gold nanoparticles investigated was taken up by the A549 (A and B) and by the NCIH441 (C and D) cells at 4°C, as shown by TEM, suggesting that particles are internalized by active endocytosis. Size bar: 1 μm.

The sample AuS0302-RIT, compared to AuS0302-RIS02 and AuS0302-RIS04 gold nanoparticles with an excess of sodium citrate, induced a mild toxicity, as observed by MTT and Ki-67 assay, and induced a lower LDH release in the cell lines A549 and NCIH441. To investigate if the reduced cytotoxicity of AuS0302-RIT was due to a defective internalization of the particles, confluent A549 and NCIH441 monolayers were incubated for 3 hr in the presence of AuNPs (0.3 mM and 1 mM in complete cell culture medium). At the end of the exposure time, cells were fixed and then analyzed by transmission electron microscopy. Figure 5 shows the comparison between the internalization of the gold nanoparticles AuS0302-RIT and AuS0302-RIS02 under different cell culture conditions. At 37°C, AuS0302-RIT nanoparticles were internalized by both A549 (Figure 5E) and NCIH441 (Figure 5I) cell lines, and the pattern was comparable to the uptake of the gold nanoparticles AuS0302-RIS02 (Figure 5G and 5K). In addition, the internalized gold particles were observed in membrane-bound vesicles (Figure 5F, H, J and 5L), mostly localized in the perinuclear region. Freely dispersed nanoparticles in the cytosol were not detected, even when cells were exposed to AuS0302-RIS04 nanoparticles (data not shown). Using an analogous experimental procedure, cells were exposed to ice-cold gold nanoparticles (0.3 mM and 1 mM solutions in complete cell culture medium) and incubated for 3 hr at 4°C. The absence of AuS0302-RIT (Figure 5A and 5C), AuS0302-RIS02 (Figure 5B and 5D) and AuS0302-RIS04 (data not shown) uptake in the cell lines A549 and NCIH441 at 4°C suggests that gold nanoparticles are internalized by active endocytosis. These observations on the intracellular localization of AuNPs are in contrast with Rothen-Rutishauser et al. [32], who demonstrated that in a cell culture model of the airway epithelium gold nanoparticles were found freely dispersed in the cytoplasm, and occasionally in the nuclei, rather than localized in membrane-bound vesicles as observed for titanium dioxide particles. However, our results are consistent with previous data showing that in human dermal fibroblasts [25], leukemia cells [26], human hepatocellular cells [30], rat alveolar macrophages and epithelial type-I cells [33], and in mouse dendritic cells and fibroblasts [34], gold nanoparticles are internalized and stored in endocytic vesicles. The uptake of gold nanoparticles seems to depend on the surface structure of NPs. In fact, water-soluble gold nanoparticles coated with ordered or disordered amphiphilic ligand shells were taken up differently by mouse dendritic cells (DC2.4 cell line) [34]. Verma et al. [34] have shown that AuNPs coated with ordered striated anionic and hydrophobic groups were internalized without disrupting the membrane integrity and particles were observed freely dispersed in the cytosol. On the contrary, gold particles coated with the same anionic and hydrophobic ligands, but randomly distributed over the particle surface, were stored in endosomal/lysosomal compartments. From our TEM study we observed that AuNPs were easily taken up by the cell lines A549 and NCIH441, and the uptake was independent of the presence or absence of contaminants on the particle surface. In addition, we observed a comparable internalization of AuNPs, although they had a relatively small size (9.5 – 11.2 – 25 nm). These data are in contrast with those made by Chithrani and Chan [35] in HeLa cells. They investigated the uptake of gold particles with a size range of 14 – 100 nm, and they concluded that the better uptake was observed for particles with a diameter of 50 nm. In fact, Chithrani and Chan have quantitated the uptake of AuNPs in HeLa cells and they demonstrated that, in the experimental conditions they applied, small particles (14 nm), as well as larger NPs (100 nm), are poorly internalized. Electron microscopy images clearly show that in HeLa cells both 14 and 100 nm AuNPs were internalized in cytoplasmic vesicles and they appeared monodispersed. On the contrary, 30 nm and 50 nm particles appeared to agglomerate. Nevertheless, the aim of our study was to investigate if the surface coating influences the uptake of AuNPs in ATII-like cells. From the observations we made it is clear that even if toxicity seems to depend on the surface contaminants, the uptake of AuNPs is not affected by the presence or absence of sodium citrate on the particle surface.

## Conclusion

In conclusion, our results suggest that the presence of contaminants, such as sodium citrate, affects the viability and the proliferation of the human alveolar type-II-like cell lines A549 and NCIH441 *in vitro*, rather than the size of the particles. Dialyzed and purified gold nanoparticles could induce a milder cytotoxicity in A549 and NCIH441 cells compared to the particles with an excess of sodium citrate. In addition, the absence of contaminants does not influence the uptake of gold particles. Our data support the fact that the human ATII-like cell lines A549 and NCIH441 internalize gold NPs by active endocytosis independent of the size and of the presence or absence of contaminants in solution. Moreover, the uptake can be inhibited by exposing the cells to low temperature. Further studies are underway to elucidate the uptake mechanism and the intracellular fate of gold nanoparticles.

## Methods

### Synthesis and characterization of gold nanoparticles

Gold nanoparticles (AuNPs) were synthesized in water by reducing tetrachloroauric acid (HAuCl_4_, purchased by Colorobbia S.p.A., Italy) in a sodium citrate solution containing polyvinylpirrolidone (PVP; (C_6_H_9_NO)_n_) as stabilizing agent [23,36]. To produce nanoparticles which retain their physicochemical properties over time and do not aggregate, the wet chemical synthesis of AuNPs via reduction of gold salts was employed [37]. A water solution of 0.5% (w/w) PVP, purchased by Toscochimica (Prato, Italy), was heated to 60°C and HAuCl_4_, characterized by a gold content of 30%, as determined by inductively coupled plasma-atomic emission spectrometry (ICP-AES), was added to obtain a final gold concentration of 0.2% (w/w). To this intermediate compound, an aqueous solution of sodium citrate (16.5% w/w) was added and the resulting product was kept at 60°C for 1 hr and afterwards air-cooled to room temperature. The resulting sample was named AuS0302 and was characterized by the presence of an excess of sodium citrate and a particle diameter of 9.5 nm as shown by STEM images. A second series of gold nanoparticles with larger average size (samples AuS0302-RIS02 and AuS0302-RIS04) were prepared as follows: to synthesize AuS0302-RIS02, AuS0302 was used as initial seed (25% of the total gold content) and mixed with PVP. The solution was heated to 60°C and suitable amounts of tetrachloroauric acid were added to maintain the final gold concentration at 0.2% (w/w). As final step, sodium citrate was added and the solution kept at 60°C, prior to cooling to RT. The batch AuS0302-RIS04 was prepared using a similar procedure, but the compound AuS0302-RIS02 was used as seed for this synthesis. To remove excess of sodium citrate from the sample AuS0302, tangential flow dialysis was performed using an Amicon system (Millipore) equipped with Biomax membrane (cut-off 10.000 KDa) (Millipore). The total volume dialysed was 36 times the initial volume and the resulting samples, which had only about 8% sodium citrate on the particle surface, was termed AuS0302-RIT.

### Cell culture and Cytotoxicity assays

#### Cell culture

The human alveolar type-II (ATII)-like cell lines A549 (ATCC number CCL-185,) and NCIH441 (ATCC number HTB-174) were purchased by LGC Promochem (Wesel, Germany). Cells were cultured in complete cell culture medium composed of RPMI 1640 with L-Glutamine (Invitrogen Corporation, Germany) supplemented with 10% (v/v) Fetal Bovine Serum (Sigma Aldrich, Germany) and 1% (v/v) 10000 U/ml Penicillin and 10000 U/ml Streptomycin (Invitrogen Corporation, Germany). Cells were maintained under standard cell culture conditions (5% CO_2_, 95% humidity and 37°C in HERAEUS incubators, Germany) and passaged weekly.

#### Cytotoxicity assays

The cytotoxicity induced by gold nanoparticles was investigated by 3-(4,5-dimethylthiazol-2-yl)-2,5-diphenyltetrazolium bromide (MTT) assay, Ki-67 assay and lactate dehydrogenase (LDH) release assay. All tests were performed using sub-confluent cells in the logarithmic growth phase. Cells (4.0 × 10^4 ^A549 cells/ml and 1.5 × 10^5 ^NCIH441 cells/ml; 100 μl/well in complete cell culture medium) were seeded in 96 well plates (TPP, Switzerland). Cells were then exposed for 24, 48 and 72 hr to concentrations of gold nanoparticles ranging from 0 mM to 0.7 mM. Nanoparticles were dispersed in complete cell culture medium RPMI 1640 with L-Glutamine (Invitrogen Corporation, Germany), with 5% Fetal Bovine Serum (Sigma Aldrich, Germany) and 1% (v/v) 10000 U/ml Penicillin and 10000 U/ml Streptomycin (Invitrogen Corporation, Germany). For each gold nanoparticle a blank solution was tested and no cytotoxicity could be observed. A 10% DMSO solution was used as positive control. Three independent experiments and 3 replicates for each experiment were performed.

In the MTT assay, after 60 hr incubation cell culture medium was replaced with fresh medium containing AuNPs at concentrations ranging from 0 mM to 0.7 mM. After 24, 48 and 72 hr continuous exposure to AuNPs, medium was removed, cells were washed with PBS and then incubated with cell culture medium containing 20% MTT solution (stock solution 5 mg MTT/ml PBS; Sigma Aldrich, Germany). After 3 hr incubation at 37°C and 5% CO_2_, 100 μl lysis buffer (20 g SDS dissolved in 50 ml ddH_2_O and supplemented with 50 ml N, N-Dimethylformamide. pH 4.7) per well was added. Cells were further incubated overnight and the absorbance was measured by spectrophotometry at λ_1 _= 620 nm and λ_2 _= 750 nm. Results were analyzed as the average of viability (% of the untreated control) ± Standard Deviation (SD).

For the Ki-67 assay, sub-confluent cells were exposed to gold nanoparticles for 24–72 hr, fixed using 1% (v/v) methanol/ethanol solution and washed five times in PBS. Fixed cells were permeabilized with 0.1% Triton-x 100 (v/v) in PBS, and after 10 minutes of permeabilization at room temperature, cells were washed three times in a solution containing PBS/0.05% Tween-20 (v/v) and incubated (45 minutes at 37°C on a rotating plate) with 1 μg/ml mouse anti-human Ki-67 antibody (clone MIB-1; DAKO, Germany). Cells were then incubated for 45 minutes at 37°C with anti-mouse IgG1 peroxidase (DAKO, Germany), and washed with PBS/0.05% Tween-20. Afterwards cells were incubated 10–20 minutes at 37°C with a solution (170 μl/well) composed by 5 mL 10× citric buffer mixed with 45 mL water, 20 μL 30% oxygen peroxide and *o*-Phenylenediamine. The solution was pipetted in a new 96 well plate containing 3 M chloridric acid (HCl) (50 μl/well), and the fluorescence was detected by spectrophotometry at 492 nm. Results are expressed as the mean value of cellular proliferation (% of the untreated control) ± Standard Deviation (SD).

In the LDH assay (Promega Corporation, Germany) 50 μl/well supernatant, collected after exposing the cell lines A549 and NCIH441 to AuNPs, were incubated with an equivalent volume of substrate solution. After incubating the plate for 30 minutes at RT, 50 μl/well stop solution was pipetted and data were acquired by spectrophotometry at 490 nm. Results are expressed as mean LDH release (expressed as experimental LDH release at 490 nm/maximum LDH release at 490 nm ± Standard Deviation (SD).

### Uptake and transmission electron microscopy studies

The human ATII-like cell lines A549 and NCIH441 were seeded on fibronectin-coated Thermanox cover slips (NUNC – Thermo Fisher Scientific, Germany) and cultivated until confluent. For the incubation at 37°C, cells were exposed to 0.3 mM and 1 mM gold nanoparticles AuS0302-RIT, AuS0302-RIS02 and AuS0302-RIS04. For the incubation at 4°C, cells were first pre-cooled for 30 minutes at 4°C, and then exposed to ice-cold AuNPs in the same concentration as for the 37°C test. For both cell incubation conditions, after 3 hr exposure to AuNPs cells were fixed in 3.7% (v/v) paraformaldehyde in PBS (20 min at RT). Afterwards cells were prepared for transmission electron microscopy analysis as follows: cells were fixed in 1% (w/v) osmium tetroxide for 2 hr and dehydrated in ethanol. Cells were passaged through propylene oxide, then samples were embedded in agar-100 resin (PLANO, Germany) and polymerized at 60°C for 48 hr. Ultrathin sections were cut with an ultramicrotome (Leica Microsystems, Germany), placed onto copper grids and stained with 1% (w/v) uranyl acetate in alcoholic solution and lead citrate. Ultrastructural analysis and photomicroscopy were performed with a transmission electron microscope EM 410 (Philips; Eindhoven, Netherlands).

## Competing interests

The authors declare that they have no competing interests.

## Authors' contributions

Gold nanoparticles were designed, synthesized and characterized by DB, GL and GB. MIH, CP, REU and CJK contributed to the interpretation of data; REU and CJK have been also involved in revising the manuscript. CUB contributed by conceiving the study, acquiring, analyzing and interpreting the results, and prepared the manuscript. All the authors read and approved the final manuscript.

## References

[B1] Oberdorster G, Ferin J, Lehnert BE (1994). Correlation between particle size, in vivo particle persistence, and lung injury. Environ Health Perspect.

[B2] Nel A, Xia T, Madler L, Li N (2006). Toxic potential of materials at the nanolevel. Science.

[B3] Oberdorster G, Ferin J, Gelein R, Soderholm SC, Finkelstein J (1992). Role of the alveolar macrophage in lung injury: studies with ultrafine particles. Environ Health Perspect.

[B4] Tran CL, Buchanan D, Cullen RT, Searl A, Jones AD, Donaldson K (2000). Inhalation of poorly soluble particles. II. Influence Of particle surface area on inflammation and clearance. Inhal Toxicol.

[B5] Han G, Ghosh P, Rotello VM (2007). Multi-functional gold nanoparticles for drug delivery. Adv Exp Med Biol.

[B6] Han G, Ghosh P, Rotello VM (2007). Functionalized gold nanoparticles for drug delivery. Nanomed.

[B7] Jain KK (2007). Applications of nanobiotechnology in clinical diagnostics. Clin Chem.

[B8] Colvin VL (2003). The potential environmental impact of engineered nanomaterials. Nat Biotechnol.

[B9] Gehr P, Bachofen M, Weibel ER (1978). The normal human lung: ultrastructure and morphometric estimation of diffusion capacity. Respir Physiol.

[B10] Oberdorster G, Oberdorster E, Oberdorster J (2005). Nanotoxicology: an emerging discipline evolving from studies of ultrafine particles. Environ Health Perspect.

[B11] Donaldson K, Tran CL (2002). Inflammation caused by particles and fibers. Inhal Toxicol.

[B12] Nel A (2005). Atmosphere. Air pollution-related illness: effects of particles. Science.

[B13] Stone V, Johnston H, Clift MJ (2007). Air pollution, ultrafine and nanoparticle toxicology: cellular and molecular interactions. IEEE Trans Nanobioscience.

[B14] Donaldson K, Stone V, Tran CL, Kreyling W, Borm PJ (2004). Nanotoxicology. Occup Environ Med.

[B15] Elder A, Gelein R, Silva V, Feikert T, Opanashuk L, Carter J, Potter R, Maynard A, Ito Y, Finkelstein J (2006). Translocation of inhaled ultrafine manganese oxide particles to the central nervous system. Environ Health Perspect.

[B16] Oberdorster G, Sharp Z, Atudorei V, Elder A, Gelein R, Lunts A, Kreyling W, Cox C (2002). Extrapulmonary translocation of ultrafine carbon particles following whole-body inhalation exposure of rats. J Toxicol Environ Health A.

[B17] Geiser M, Rothen-Rutishauser B, Kapp N, Schurch S, Kreyling W, Schulz H, Semmler M, Im Hof V, Heyder J, Gehr P (2005). Ultrafine particles cross cellular membranes by nonphagocytic mechanisms in lungs and in cultured cells. Environ Health Perspect.

[B18] Muhlfeld C, Geiser M, Kapp N, Gehr P, Rothen-Rutishauser B (2007). Reevaluation of pulmonary titanium dioxide nanoparticle distribution using the "relative deposition index": Evidence for clearance through microvasculature. Part Fibre Toxicol.

[B19] Peters A, Veronesi B, Calderon-Garciduenas L, Gehr P, Chen LC, Geiser M, Reed W, Rothen-Rutishauser B, Schurch S, Schulz H (2006). Translocation and potential neurological effects of fine and ultrafine particles a critical update. Part Fibre Toxicol.

[B20] Nemmar A, Hoet PH, Vanquickenborne B, Dinsdale D, Thomeer M, Hoylaerts MF, Vanbilloen H, Mortelmans L, Nemery B (2002). Passage of inhaled particles into the blood circulation in humans. Circulation.

[B21] Rosi NL, Giljohann DA, Thaxton CS, Lytton-Jean AK, Han MS, Mirkin CA (2006). Oligonucleotide-modified gold nanoparticles for intracellular gene regulation. Science.

[B22] Podsiadlo P, Sinani VA, Bahng JH, Kam NW, Lee J, Kotov NA (2008). Gold nanoparticles enhance the anti-leukemia action of a 6-mercaptopurine chemotherapeutic agent. Langmuir.

[B23] Daniel MC, Astruc D (2004). Gold nanoparticles: assembly, supramolecular chemistry, quantum-size-related properties, and applications toward biology, catalysis, and nanotechnology. Chem Rev.

[B24] Goodman CM, McCusker CD, Yilmaz T, Rotello VM (2004). Toxicity of gold nanoparticles functionalized with cationic and anionic side chains. Bioconjug Chem.

[B25] Pernodet N, Fang X, Sun Y, Bakhtina A, Ramakrishnan A, Sokolov J, Ulman A, Rafailovich M (2006). Adverse effects of citrate/gold nanoparticles on human dermal fibroblasts. Small.

[B26] Connor EE, Mwamuka J, Gole A, Murphy CJ, Wyatt MD (2005). Gold nanoparticles are taken up by human cells but do not cause acute cytotoxicity. Small.

[B27] Shukla R, Bansal V, Chaudhary M, Basu A, Bhonde RR, Sastry M (2005). Biocompatibility of gold nanoparticles and their endocytotic fate inside the cellular compartment: a microscopic overview. Langmuir.

[B28] Pan Y, Neuss S, Leifert A, Fischler M, Wen F, Simon U, Schmid G, Brandau W, Jahnen-Dechent W (2007). Size-dependent cytotoxicity of gold nanoparticles. Small.

[B29] Niidome T, Yamagata M, Okamoto Y, Akiyama Y, Takahashi H, Kawano T, Katayama Y, Niidome Y (2006). PEG-modified gold nanorods with a stealth character for in vivo applications. J Control Release.

[B30] Gannon CJ, Patra CR, Bhattacharya R, Mukherjee P, Curley SA (2008). Intracellular gold nanoparticles enhance non-invasive radiofrequency thermal destruction of human gastrointestinal cancer cells. J Nanobiotechnology.

[B31] Hauck TS, Ghazani AA, Chan WC (2008). Assessing the effect of surface chemistry on gold nanorod uptake, toxicity, and gene expression in mammalian cells. Small.

[B32] Rothen-Rutishauser B, Muhlfeld C, Blank F, Musso C, Gehr P (2007). Translocation of particles and inflammatory responses after exposure to fine particles and nanoparticles in an epithelial airway model. Part Fibre Toxicol.

[B33] Takenaka S, Karg E, Kreyling WG, Lentner B, Moller W, Behnke-Semmler M, Jennen L, Walch A, Michalke B, Schramel P (2006). Distribution pattern of inhaled ultrafine gold particles in the rat lung. Inhal Toxicol.

[B34] Verma A, Uzun O, Hu Y, Hu Y, Han HS, Watson N, Chen S, Irvine DJ, Stellacci F (2008). Surface-structure-regulated cell-membrane penetration by monolayer-protected nanoparticles. Nat Mater.

[B35] Chithrani BD, Ghazani AA, Chan WC (2006). Determining the size and shape dependence of gold nanoparticle uptake into mammalian cells. Nano Lett.

[B36] Xiong Y, Washio I, Chen J, Cai H, Li ZY, Xia Y (2006). Poly(vinyl pyrrolidone: a dual functional reductant and stabilizer for the facile synthesis of noble metal nanoplates in aqueous solutions. Langmuir.

[B37] Jana NR, Gearheart L, Murphy CJ (2001). Evidence for seed-mediated nucleation in the chemical reduction of gold salts to gold nanoparticles. Chem Mater.

